# Harvested wood products and REDD+: looking beyond the forest border

**DOI:** 10.1186/s13021-016-0046-9

**Published:** 2016-05-21

**Authors:** Tunggul Butarbutar, Michael Köhl, Prem Raj Neupane

**Affiliations:** 1 GIZ Forest and Climate Change Program, Manggala Wanabakti Bd. Block VII Fl. 6, Jl. Gatot Subroto, Jakarta, 10270 Indonesia; 2 University of Hamburg, World Forestry, Leuschnerstr. 91, 21031 Hamburg, Germany; 3 SURF, Leuschnerstr. 91, 21031 Hamburg, Germany

**Keywords:** REDD+, HWP, Material substitution, Energetic substitution, Sustainable forest management, Emission reductions, Displacement factor

## Abstract

**Background:**

The focus of REDD+ is sensu stricto on maintaining forest carbon stocks. We extend the scope of sustainable management of forest from forests to timber utilization, and study carbon offsets resulting from the utilization of harvested timber for bio energy or harvested wood products (HWPs). The emission budget of harvesting operations depends on the loss of standing biomass by timber extracted from the forest site and logging losses on the one side, and on the other on the wood end use and the utilization of processing residues. We develop two scenarios to quantify the magnitude of CO_2_ emissions by (1) energetic utilization, and (2) energetic and material utilization of harvested timber and compare the substitution effects for different fossil energy sources.

**Results:**

The direct energetic use of harvested timber does not compensate for the losses of forest carbon stock. Logging residuals and displacement factors reflecting different wood use constitute by far the most important factor in potential emission reductions. Substitution effects resulting from energetic use of mill residuals and from HWPs have only a subordinated contribution to the total emissions as well as the type of fossil fuel utilized to quantify substitution effects. Material substitution effects associated with harvested wood products show a high potential to increase the climate change benefits.

**Conclusions:**

The observation and perception of REDD+ should not be restricted to sustainable management and reduced impact logging practices in the forest domain but should be extended to the utilization of extracted timber. Substitution effects from material and energetic utilization of harvested timber result in considerable emission reductions, which can compensate for the loss of forest carbon, and eventually contribute to the overall climate change mitigation benefits from forestry sector.

## Background

Forests provide a multitude of ecosystem services and functions, among which are their role in the global carbon cycle, the supply with timber and fuel wood, or safeguarding biodiversity. The current promotion of bio-economy and the related extension of renewable energies are likely to increase the demand for timber. Decisions about the appropriate management and utilization of forests create a vigorous area that is fueled by differences in social, cultural, environmental and ecological aspects concerning “optimal” forest management and utilization strategies for enhancing the contribution of forests to the mitigation of climate change.

Forest related options for mitigating climate change include the sequestration of atmospheric carbon dioxide (CO_2_) by forest growth, the conservation and enhancement of forest carbon stocks as well as the substitution, and C-storage resulting carbon effects from the utilization of harvested timber. This offers three ways for treating forest carbon stock in order to achieve mitigation: (i) maintaining and enhancing forest biomass stock and avoiding emissions from forest degradation and deforestation, (ii) use as a renewable source of energy (bioenergy) for substitution of fossil fuels, or (iii) use as renewable material (harvested wood products, HWPs) for substitution of alternative products and materials, production of which is associated with higher energy consumption and thus emissions.

In the Kyoto Protocol’s second commitment period (2013–2020) [1], C-stock changes in the HWPs pool are explicitly included in the calculation of the country’s greenhouse gas (GHG) emissions and removals. The reducing emissions from deforestation and forest degradation (REDD+) mechanism, which has been under negotiation by the United Nations Framework Convention on Climate Change (UNFCCC) since 2005, focuses on activities that developing countries may implement to reduce emissions and enhance removals of greenhouse gases. Five “eligible activities” have been defined under REDD+ [2]:

- Reducing emissions from deforestation;
- Reducing emissions from forest degradation;
- Conservation of forest carbon stocks;
- Sustainable management of forests; and
- Enhancement of forest carbon stocks.

The formal and strong recognition of the role of forests mitigating climate change and the explicit recognition of REDD+ as a mechanism to contribute to reducing emissions and enhancing carbon sinks in Article 5 of Paris Agreement encouraged parties, particularly developing countries, to reduce carbon emissions, and conserve and sustainable management of their standing forests. The universal and landmark climate deal also calls on parties to adhere already agreed REDD+ related COP decisions of the Conference of the Parties (COP) to the United Nations (Article 5.2). Along with such international policy developments and involvements, research into carbon balancing pertaining to HWPs and consideration of relevant climate change mitigation strategies are increasingly growing [3].

The focus of REDD+ is sensu stricto on maintaining forest carbon stocks. Measurement, reporting and verification in the scope of REDD+ are related to carbon released from and carbon sequestered by forests. Under REDD+ every carbon removals from the managed forest area are considered as emissions; whereas the long-lived carbon storage by harvested wood products or material substitution effects induced by the use of timber instead of non-renewable resources is not accounted for. HWP so far is part of the national GHG-reporting, but not considered in REDD+. However, the Paris Agreement strongly encourages all parties to consider the entire sinks and reservoirs of greenhouse gas while developing the nationally appropriate mitigations actions, pathways to implement the agreement, and policy approaches [4].

We extend the scope of sustainable management of forest from forests to timber utilization, and study carbon offsets resulting from the utilization of harvested timber for bioenergy or HWPs. We develop scenarios to quantify the magnitude of CO_2_ effect in different uses of HWP and elaborate on the potential impact on emission reduction accounting under REDD+ in a future (post 2015) international treaties.

### Contribution of harvested wood products (HWPs) to climate change mitigation

The “Revised 1996 IPCC Guidelines” recommend a default approach under which all CO_2_ emissions and removals associated with forest harvesting and the oxidation of wood products are accounted for by the country in the year of harvesting (i.e., removal from the forest biomass pool). This approach laid the foundations for the widely shared supposition that the use of timber is carbon neutral. However, there is no common understanding of the term “carbon neutrality”. Treating harvested timber as carbon neutral is only justified when the loss of carbon from the forest C-stocks has already been accounted for at the time of harvest.

Following the IPCC Guidelines for National Greenhouse Gas Inventories [5] carbon contained in harvested timber can be transferred from the forest C-pool to the C-pool of HWPs. Under this approach burning of timber would result in CO_2_ emissions. These CO_2_ emissions can be compared to fossil fuel emissions for producing a unit amount of energy in order to see whether the use of timber results in an emission reduction. However, this direct comparison does not take into account the release of carbon content from biomass decay to the atmosphere, regardless of whether it is utilized or not.

HWPs contribute to the climate change mitigation in three ways: (i) carbon storage effect, (ii) material substitution effect, and (iii) energy substitution effect. Wood fuel can be used as a renewable source of energy to substitute fossil fuels, which reduces additional CO_2_ emissions to the global carbon cycle, as the combustion of wood fuel releases only carbon that is already part of the global carbon balance. This energy path can contribute to renewable energies in different forms: (i) energy provision directly from wood, (ii) bioenergy production from logging and processing residues, and (iii) use of the wood contained in HWPs for energy production at the end of their lifecycle [6–8]. In 2011, for wood fuel 1343 million m^3^ of harvested timber were used [9].

Timber as renewable material allows for the physical storage of carbon and for producing wooden products. According to Maraseni [10, 11] carbon is locked for another 46 years in HWPs. Wooden products are compared to alternative materials of equal functionality generally associated with lower energy input in the production process. Studies show, for example, that substitution of CO_2_ and energy intensive materials (steel, alloys, concrete) by wood is associated with substantially lower emissions of CO_2_ [12–14]. Moreover, a substantial reduction in the consumption of fossil fuels in the production and transportation of high energy-consuming materials can be realized [15]. Thus, the analysis of forestry contribution to climate change mitigation should take to account the important role of HWPs [16, 17]. Emission reductions per unit biomass can generally be enhanced if material substitution effects and energy substitution effects attributed to HWPs are combined. This can be realized when the timber contained in HWPs is used for energy at the end of the life-cycle of the product [18]. In addition, HWPs can be recycled and used in successive products. The so-called cascade use of HWPs has a successive potential for emission reduction.

### Approaches for emission accounting

**Fig. 1:**
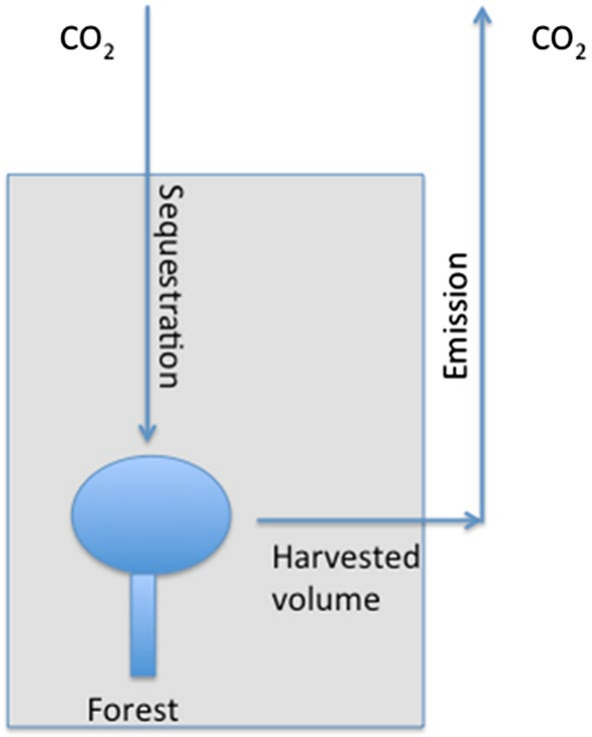
The current HWP position under REDD+ carbon dynamic

Current discussions of the REDD+ mechanism give a major priority to C-stock losses in forests induced by forest degradation and deforestation. This is justified in situations where REDD+ is seen as an instrument to maintain and enhance forest carbon stocks and thus any degradation and deforestation activity is to be treated as emission (Fig. 1).

However, timber harvesting can be seen as a transition of carbon from the forest carbon pool to the harvested wood products pool [19–21]. For Annex I countries the second commitment period of the Kyoto Protocol (2013–2020) explicitly allows for the consideration of the C-stock and C-stock changes in HWPs pools. This calls for a revision of the concept of “carbon neutrality” of HWPs and wood fuel. The carbon contained in harvested biomass is no longer treated as direct emission from the forest C-stock to the atmosphere at the time of harvesting. HWPs serve as an intermediate C-stock and any combustion of timber, either of HWPs at the end of their lifetime or of wood fuel, is regarded as emission.

As the underlying processes and interrelationships are complicated, much attention is given to consistent and transparent accounting rules [15]. Any accounting rule under the UNFCCC is the result of a consensus between different actors, and has to take into account the higher order set of rules and regulations. What allows for consistent and reliable national GHG-reporting is negotiated and implemented for different sectors. C-stock changes in forests and HWPs are accounted for in the LULUCF/forestry sector, while emissions from energy are accounted for in the energy supply sector. This hampers a direct link to emission reductions associated to the forest-timber chain. Compared to alternative materials with similar function, HWPs generally show lower energy consumption and emissions in their production processes. In national GHG inventories, these emission reductions are accounted for in the energy sector. Therefore, it might be advisable to decouple the general reflection of the mitigation potential of HWPs from the UNFCCC accounting framework.

### Energetic use

When studying the energy substitution effect of timber, it is crucial to consider which type of fossil fuel is compared. Our comparison follows a study conducted by [22] and utilizes natural gas, lignite, and residual fuel oil as references for energetic substitution (Box 1). Table 1 presents the net caloric values (TJ/Gg) and CO_2_ emission factors (kg/MWh) associated with different types of fossil fuels. The presented effective CO_2_ emission factors are default values taken from [5]. The IPCC default values assume dry matter biomass and are considerably lower than those given by other authors (e.g., http://www.engineeringtoolbox.com).

**Table 1:** Default values for net caloric value, effective CO_2_ emission factors (Source: IPCC 2006), and life cycle CO_2_ emissions (Source: http://www.biomassenergycentre.org.uk)

Energy source	Net caloric value (TJ/Gg)	Effective CO_2_ emissions (kg/MWh)	Life cycle CO_2_ emissions (including production) (kg/MWh)
Lignite	11.9	364	414
Residual fuel oil	40.4	279	314
Natural gas	48.0	202	227
Wood/wood waste	15.6	403	403^a^

^a^Assuming utilization for subsistence with only manual interventions

Direct or effective emissions account for the emissions associated with energy combustion, but do not account for emissions arising from manufacturing, infrastructure or transport associated with energy technologies and fuels [23]. Indirect emissions are a consequence of the activities that occur at sources controlled by other entities than the end user and comprise all the emissions from the final use back to raw material extraction. Life cycle CO_2_ emissions combine direct and indirect emissions and depend strongly upon details of supply chains, production techniques, forestry practices, or transport distances [23, 24]. The life-cycle analysis can adopt different analytical methodologies and are affected by data availability and uncertainties surrounding the value of key attributes. This holds especially true for life-cycle analysis carried out in developing countries [25]. For the current study, we utilize values presented by the biomass energy centre (http://www.biomassenergycentre.org.uk) in order to approximate life cycle CO_2_ emissions.

Timber is an inhomogeneous fuel. Its caloric value depends on the content of water, cellulose, lignin, resin, acids, oils, and minerals and varies between 4.17 and 4.72 kWh per kg [28]. Decisive for the caloric value is the water content of timber. When timber is burnt firstly the water contained in timber is evaporated. In order to evaporate a kg of water contained in the wood, 0.68 kWh (2.45 MJ) energy is needed at 20 °C. For our scenario analysis we assumed air-dried timber with a water-content of 15 % and a caloric value of 4.33 kWh per kg. Caloric values are linked to tree biomass weight and volume by wood density. We selected three different wood densities (500; 750; 1000 kg/m^3^) to present the potential range of wood densities found in tropical tree species.

When timber is used to replace natural gas, lignite, or residual fuel oil for energy production the respective emissions have to be compared for a unit reference. The results presented in Table 2 allow for quantifying the direct CO_2_ emissions of alternative energy sources with reference to the caloric value produced by the combustion of 1 m^3^ of timber of different wood densities. For the current study both, effective and lifecycle CO_2_ emissions were utilized with the purpose of demonstrating the sensitivity of findings with respect to imputed emission factors.

### Box 1: Reference types of fossil fuels

*Natural gas* is a naturally occurring gas mixture, which consists mainly of methane.

*Lignite* is the lowest rank of coal, often referred to as brown coal, used almost exclusively as fuel for steam-electric power generation. It is brownish-black and has high inherent moisture content, sometimes as high as 45 %. The heat content of lignite ranges from 2600 to 5000 kWh per ton on a moist, mineral-matter-free basis [26].

*Residual fuel oil* is a general classification for heavier oils that remain after the distillate fuel oils and lighter hydrocarbons are distilled away in refinery operations. It is used in steam-powered vessels in government service and inshore power plants, the production of electric power, space heating, vessel bunkering, and various industrial purposes [27].

### Emissions due to logging residues

Harvesting operations may induce pronounced reductions of the growing stock and thus forest carbon stocks. In a study conducted in Malaysian State of Sarawak, Noack [29] showed that on average about 54 % of the total above ground wood volume of trees removed from a stand was extracted in the form of logs. These findings are supported by McLeish and Sustany [30]. For tropical countries felling recovery rates related to aboveground wood volume were estimated to be 54 % in Africa, 46 % in Asia/Pacific, 56 % in Latin America and the Caribbean, and 50 % on average for all tropical areas [31, 32]. Noack [33] found, in a similar study for Ghana, Cameroon, East Kalimantan and Sarawak, that on average 53.5 % of the total extracted volume was logs of the trees those having a diameter at breast height greater than 20 cm. Of the remaining volume 4.6 % was stump, 5.2 % buttress, 10.4 % stem off-cuts and 26.3 % were parts of the crown. For Malaysia and Sri Lanka, Enters [34] showed that between 30 and 48 % of the timber of felled trees is utilized. He notes that as a “traditional rule-of-thumb” for “every cubic meter of wood extracted from the forest another is left behind”.

These figures are related to the timber extracted from felled trees. Carbon stock reductions resulting from harvesting operations include logging residues additional to non-utilized components of felled trees that remain in the forests. Additional logging residues may be caused by the felling of trees for the creation of skidding trails and road infrastructure, trees damaged or killed in connection with the felling of crop trees, or non-merchantable woody parts of crop trees that remain in the forest. Thurland [35] reported for an unsupervised logging operation in the Malaysian State of Terengganu growing stock reductions of 50–70 % to the residual stands. According to a study reported by Pearson et al. [36], the volume of logging residues in Belize, Bolivia, Brazil, Indonesia, Guyana, and Republic of the Congo is 2–5 times higher than the volume of extracted timber. The substantial variations in felling recovery rates reported are subject to operational efficiency and skill of workers, available markets for lower grade logs, or differences in the definition of merchantable wood [37]. The application of reduced impact logging is a relevant factor for recovery rates [38–40]. Logging residues inside the forest may also remain as an organic carbon. However, we applied a conservative approach by treating the logging residues as immediate emissions in order to avoid the strenuous and arduous emission benefits associated with the residues.

### Emissions due to processing residues

Processing of logs in sawmills results in final products and residues. Mill residues include woody material generated when round wood is processed into primary wood products. The composition of mill residues depends on the primary product and on processing technologies. The mill residues include among others slabs, edgings, trimmings, sawdust, or veneer clippings and cores. Plywood mills produce quite different residues than saw-mills. According to Enters [34], mill waste can be divided into bulk waste, which is made up of larger pieces, and fine wood particles, which consists of shavings, sawdust and sander dust.

The volume of mill residues is affected by numerous factors. The recovery rate in timber processing is especially dependent on log dimensions. Ravn and Jensen [41] reported that for logs in the range of 30–70 cm in diameter, recovery rates drop to about half when the log diameter is halved. Additional decisive factors for recovery rates are tree species, log quality, timber defects, sawmilling equipment, mill maintenance, production methods, grading, storage and drying [34, 42]. Enters [34] analyzed detailed studies in numerous developing countries and found sawmill recovery rates in a range from 42 to 60 % with an average of 50.8 % and plywood recovery rates in a range from 43 to 50 % with an average of 46.9 %.

### Emissions related to HWPs

According to Sathre and O’Connor [19], the “comparative analysis of the carbon balances of wood vs. non-wood products is a complex issue”. The analysis depends on the definition of the appropriate functional unit and the effective system boundaries. Functional units can be individual wood products, entire buildings or services provided by the built environment. System boundaries relate to the activity and the temporal and spatial dimension. The activity based life cycle processes include material production, product operation, and the post-use material management. Temporal system boundaries can extent from the production of the raw material, the product processing and product life-cycle, the duration of carbon storage in the product, recycling of the product, the availability of residue biofuels, and the fate of the wood product at the end of the product’s lifetime (e.g., energetic use, decay, or disposal). Therefore, life cycle analysis generally relates to specific HWPs and takes into account their entire life cycle including production, use, and disposal. Knauf et al. [6] quantified the GHG impacts of different HWPs in the regional environment of north-western Germany. The post-use of HWPs is “the single significant source of variability in the GHG impacts of the wood product life cycle” [19].

To what extent the HWPs contribute to reduce the GHG emission is a key issue while quantifying the amount to which GHG emission can be reduced by the use of forest biomass to mitigate climate change. The displacement factor is an index that quantifies the efficiency of emission reductions per unit of wood use. In a meta-analysis Sathre and O’Connor [19] found displacement factors of wood products ranging between −2.3 and 15. The use of timber in this analysis varies from construction, housing, apartment, hotel and energy. Negative displacement factors indicate that the wood products lead to greater GHG emissions than the use on non-wood products, which is mostly caused by inappropriate disposal. In general, disposal of wood products typically require less energy than products made from other high energy-consuming materials [16]. According to Sathre and O’Connor [19] the displacement factor for wood being used directly as biofuel to replace fossil fuel ranges from less than 0.5 to about 1.0, with an average value of 0.8. Based on the results of their meta-analysis, Sathre and O’Connor [19] found an average middle estimate for the displacement factor of 2.1. A displacement factor of 2.1 corresponds to 3.9 kg CO_2_e emission reduction per kg of oven-dry wood used or 1.9 t CO_2_e per m^3^ of wood product [19]. For our study we selected displacement factors of 0.8 and 2.1.

## Results and discussion

Based on a scenario approach the carbon effects of logging and mill losses as well as HWPs were studied. The results presented for the two scenarios “Wood fuel” and “HWPs” show the potential CO_2_ emission effects of the simultaneous consideration of harvesting induced losses in forest carbon stocks and substitution effects by timber utilization. Negative values in the result tables indicate that the use of timber results in higher emissions than those from utilizing the three selected non-renewable energy sources, while positive values indicate emission reductions. All values are based on a standard unit of 1 m^3^ of solid wood.

### Scenario 1 “wood fuel”

Scenario 1 assumes that all harvested timber is used as wood fuel without logging residues or with logging residues of the same amount as extracted timber. Table 2 presents the differences between CO_2_ emissions from the non-renewable energy sources (lignite, residual fuel oil, and natural gas) and timber. Both, effective emissions and lifecycle CO_2_ emissions of timber exceed the corresponding emissions of the selected non-renewable energy sources (Table 3). This holds especially true where the harvesting of wood fuel is associated with logging losses. Thus the energetic substitution effect of wood fuel generally does not compensate for forest C-stock losses.

**Table 2:** Scenario “wood fuel”: emission savings (kg CO_2_)

Logging residues	None			1 m^3^		
Wood density	500 kg/m^3^	750 kg/m^3^	1000 kg/m^3^	500 kg/m^3^	750 kg/m^3^	1000 kg/m^3^
Effective CO_2_ emissions						
Lignite	−130	−194	−259	−1048	−1570	−2094
Residual fuel oil	−314	−470	−628	−1232	−1846	−2463
Natural gas	−480	−720	−960	−1398	−2096	−2795
Lifecycle CO_2_ emissions						
Lignite	−21	−30	−41	−939	−1406	−1876
Residual fuel oil	−238	−355	−474	−1156	−1731	−2309
Natural gas	−426	−638	−851	−1344	−2014	−2686

**Table 3:** CO_2_ emissions (kg CO_2_) from combustion of 1 m^3^ of timber and corresponding alternative energy sources

Energy source	Wood density		
	500 kg/m^3^ (2.17 MWh)	750 kg/m^3^ (3.25 MWh)	1000 kg/m^3^ (4.33 MWh)
Effective CO_2_ emissions			
Lignite	788	1182	1576
Residual fuel oil	604	906	1207
Natural gas	438	656	875
Wood (1 m^3^)	874	1310	1747
Lifecycle CO_2_ emissions			
Lignite	897	1346	1794
Residual fuel oil	680	1021	1361
Natural gas	492	738	984
Wood (1 m^3^)	874	1310	1747

### Scenario 2 “harvested wood products”

Scenario 2 utilizes HWPs under two levels of efficiency (Table 4). The low efficiency sub-scenario 2a (Table 5) is characterized by substantial logging losses, a low displacement factor, and no energetic use of residues and HWPs at the end of their lifetime. Emissions savings by substitution effects associated with the use of HWP are low under this sub-scenario (displacement factor = 0.8) and do not have the ability to compensate for emissions from logging and mill residues. The displacement factor compensates roughly for the emissions originating from the decay of HWPs at the end of their lifetime.

**Table 4:** Assumptions for scenario 2—harvested wood products

Component	Low efficiency scenario (sub-scenario 2a)	High efficiency scenario (sub-scenario 2b)
Logging residuals	5 times the amount of extracted timber (conventional logging)	Same amount as extracted timber (reduced impact logging)
Mill residues	60 %, no energetic use	40 %, energetic use
Displacement factor	0.8 (corresponds to 1.48 kg CO_2_e emission reduction per kg of wood)	2.1 (corresponds to 3.9 kg CO_2_e emission reduction per kg of wood)
Proportion of HWPs for energetic use at end of lifecycle	0 %	60 %
Proportion of C-stock of HWPs emitted at end of life cycle	100 %	40 %

**Table 5:** Scenario 2a “harvested wood product (HWP), low efficiency”: emissions (kg CO_2_)

Wood density (kg/m^3^)	Emissions			Emission reduction with displacement factor = 0.8	Total emissions
	Logging residues^a^	Mill residues^b^	HWP end of lifecycle^c^		
500	−4590	−551	−367	300	−5208
750	−6880	−826	−550	449	−7807
1000	−9175	−1101	−734	599	−10,411

^a^5 m^3^

^b^60 %, no energetic use

^c^No energetic use

Sub-scenario 2b represents a high efficiency in timber utilization by adopting moderate logging losses, energetic use of residues and HWPs at the end of their lifetime, and a displacement factor of 2.1 (Table 6). Reduced impact logging and the energetic use of logging residuals and HWPs at the end of their lifetime result in substantially lower total emissions. More sophisticated utilization of timber results in higher displacement factors and thus increasing substitution effects. Compared to the low efficiency scenario the total emissions are considerably reduced and are for lignite life-cycle CO_2_ emissions almost balanced. A moderate increase of substitution effects could result in emission gains. Under the emission assumptions given for scenario 2b, a displacement factor larger than 2.2 would result in emission savings, if lifecycle CO_2_ emissions for lignite are considered. A displacement factor of 2.9 would compensate for effective CO_2_ emissions compared to natural gas as an alternative energy source. This indicates a potential to increase climate benefits through the changes in displacement factor driven by promoting and sophisticated use of wood products harvested from the domestic managed forests.

**Table 6:** Scenario 2b “harvested wood product (HWP), high efficiency”: emissions (kg CO_2_)

Wood density (kg/m^3^)	Emissions (kg CO_2_)		Emission reduction with displacement factor = 2.1 (kg CO_2_)	Substituted emissions for energy (kg CO_2_)			Total emissions (kg CO_2_)		
	Logging residues ^a^	HWP end of lifecycle		Lignite	Residual oil fuel	Natural gas	Lignite	Residual oil fuel	Natural gas
Effective CO_2_ emissions									
500	−918	−220	1170	−64	−204	−331	−122	−262	−389
750	−1376	−330	1755	−96	−306	−496	−182	−392	−582
1000	−1835	−440	2340	−128	−408	−661	−244	−523	−777
Lifecycle CO_2_ emissions									
500	−918	−220	1170	18	−146	−290	−40	−205	−348
750	−1376	−330	1755	27	−220	−434	−59	−306	−521
1000	−1835	−440	2340	36	−293	−579	−79	−408	−695

^a^1 m^3^

The results of the partial sensitivity analysis are presented in Table 7. For a reference unit of 1 m^3^ with a density of 500 kg, the effective CO_2_ emissions are calculated taking into consideration 17 factors (see Table 7). The factors were varied according to the range specified in the first column of Table 7.

**Table 7:** Results of sensitivity analysis: emissions (kg CO_2_)

Factor	Minimum	Maximum	Mean	Std. dev
Logging residues (1–5 times extracted timber)	874.0	4370.00	2622.00	1236.16
Mill residues (10–50 % of extracted timber)	87.40	437.00	262.200	123.61
Energy from mill residuals (10–50 % of mill residuals converted for energetic use)	8.74	218.50	78.660	55.28
Lignite substituting energy from mill residuals (effective CO_2_)	7.88	197.00	70.920	49.84
Oil substituting energy from mill residuals (effective CO_2_)	6.04	151.00	54.360	38.20
Gas substituting energy from mill residuals (effective CO_2_)	4.38	109.50	39.420	27.70
Lignite substituting energy from mill residuals (lifecycle CO_2_)	8.97	224.25	80.73	56.74
Oil substituting energy from mill residuals (lifecycle CO_2_)	6.80	170.00	61.20	43.01
Gas substituting energy from mill residuals (lifecycle CO_2_)	4.92	123.00	44.28	31.12
Displacement factor (1–5)	812.82	8778.46	3982.82	2139.98
Energy from HWPs at end of lifecycle (10–60 %)	43.70	417.96	214.13	115.05
Lignite substituting energy from HWPs^a^ (effective CO_2_)	39.40	425.52	193.060	103.73
Oil substituting energy from HWPs^a^ (effective CO_2_)	30.20	326.16	147.980	79.51
Gas substituting energy from HWPs^a^ (effective CO_2_)	21.90	236.52	107.310	57.65
Lignite substituting energy from HWPs^a^ (lifecycle CO_2_)	44.85	482.38	219.77	118.08
Oil substituting energy from HWPs^a^ (lifecycle CO_2_)	34.00	367.20	166.60	89.51
Gas substituting energy from HWPs^a^ (lifecycle CO_2_)	24.60	265.68	120.54	64.77

^a^HWPs 50–90 % of extracted timber

The sensitivity analysis showed the contribution of different factors on the total emission budget. Substitution effects resulting from energetic use of mill residuals and from HWPs have only a minor contribution to the total emissions as well as the type of fossil fuel utilized to quantify the substitution effect. This is in line with [43] which considers wood energy to be carbon neutral if it is originated from sustainably managed forests and processed using proper technology. Similarly, it plays a subordinated role if effective or lifecycle CO_2_ emissions are considered. Logging residuals and displacement factor constitute by far the most important factor in potential emission reductions. As a consequence logging residuals and the type of wood use expressed by the displacement factor are driving the benefits from REDD+ in a holistic emission budget.

Numerous studies have shown [6, 44] the potential role of HWP for emission reduction by both replacement of fossil fuels as source of energy as well as replacement of material that is associated with high emissions in the production process [19]. In the scope of REDD+ where emissions from deforestation and forest degradation are to be reduced, the carbon storage effect and material substitution effect attributed to HWPs can be substantial components to compensate for losses of forest carbon stocks, and consequently, to increase the climate change mitigation benefits substantially. Maraseni and Cockfield [11] compare the economic returns from three land use options, i.e., ‘carbon’ plantation (*Corymbia citriodora* subspecies Variegata) which includes value of carbon stored in harvested wood products, pasture, and cultivation of peanut-maize in the Kingaroy area of Queensland. The study found that the ‘carbon’ plantations are the most profitable land use option.

Logging residues cause direct CO_2_ emissions to the atmosphere. Reducing logging residues is of uttermost importance. Griscom et al. [45] report potential emission savings of 30–50 % by the adoption of reduced-impact logging. However, where logging residues are used for energy the nutrient balance of pristine forest stands has to be carefully monitored [46]. Trade-offs relationships should be investigated between the carbon storage (carbon in forests, carbon in dead organic matter and soil) and energy substitution (increasing energy generation from the logging residues) effects attributable to HWPs.

The direct energetic use of harvested timber does not compensate for the losses of forest carbon stock, while material substitution effects by HWPs result in considerable emission reductions. Innovative wood technologies can improve the substitution effects considerably and should become a substantial component in improving the mitigation potential of HWPs. Emission reductions can be further increased if mill residues and HWPs at the end of the lifetime are not used for energy but are further converted into timber products [47].

Though, the climate change mitigation benefits generated by the harvested wood products are not directly linked with and explicitly covered by the five REDD+ activities outlined by the UNFCCC, it is strongly linked with the clean development mechanism (CDM) and joint implementation (JI) mechanism under the Kyoto Protocol, and with the voluntary carbon market. Considering the emission reduction potentials of the material substitution effect associated with the harvested wood products, our study strongly recommends this missing carbon pool should be fully realized and included under the extended REDD+ mechanism. However, caution should be taken to accommodate the uncertainty and complexity while developing forest reference level, and credible, reliable and applicable MRV system for REDD+ mechanism.

However, these findings do not take into account the growth of forests after logging interventions. From managed forests it is widely known that moderate growing stock reductions by thinning stipulate the growth of the remaining stand. The remaining stand compensates higher emissions of wood fuel by a woody biomass increment between 0.09 (Lignite, no logging residues) and 1.43 m^3^ (natural gas, 1 m^3^ logging losses). Under most tropical forest conditions those increments can be realized under sustainable forest management regimes within 1 year [48].

## Conclusions

A final answer to the role of HWPs in the scope of REDD+ can only be found if the framework for CO_2_ considerations is clearly defined. If one confines any observations to the forest carbon stock, any utilization will result in carbon loss and CO_2_ emissions. Where the focus is on the global carbon cycle, shifts between carbon pools and the resulting change of their sizes are considered. Under these conditions the utilization of timber is a mere shift within the system, while any utilization of fossil fuels will result in an increase of the total amount of carbon in the system. The negative effects of increasing atmospheric carbon are widely known. Thus REDD+ will have a positive contribution to emission reductions only, if on one hand the harvested timber is used to substitute emissions from fossil fuels, and, on the other hand, the time lag between reductions of the forest carbon stock due to logging and the release of the respective carbon to the atmosphere can be extended in time, in a way that the remaining forest stock has enough time to compensate for carbon losses by carbon sequestration due to forest growth.

Under the scenario considering all harvested timber is used as wood fuel, CO_2_ emissions of timber exceed the corresponding emissions of the selected non-renewable energy sources. This implies that the energy substitution effects associated with the harvested wood products by the direct energetic use of timber does not compensate for the loss in forest carbon stock.

This poses a particular problem in forests where the procurement of wood fuel is the driving factor of forest degradation and deforestation. As 1343 Mio m^3^ or 80 % of the global timber harvest in 2011 was utilized for energy [9] the problem is particularly clear. The utilization of harvested wood as well as the improvement of harvesting systems play a decisive role in the carbon dynamics in the entire lifecycle of forest carbon. In regard to the material substitution effects associated with the HWPs, the study shows potentials to increase the climate change mitigation benefits by reducing logging residues and through the increase in displacement factors driven by innovative wood technologies, and promoting and sophisticated use of harvested wood products.

Wherever forests are deforested and converted to other land-use the incidental growing stock needs to be utilized. In Africa and South America alone deforestation involves an estimated growing stock of almost 500 Mio m^3^.

The analysis of forestry contribution to climate change mitigation renders accounting for the essential role of HWPs is necessary [12]. Holistic and integrative approaches combining the reduction of emissions from logging, efficiency in biomass use as well as the efficient use of HWPs are to be implemented as policy measures. This renders a systemic approach necessary that links emissions from timber extraction in the agriculture, forest and land-use sector (AFOLU) with emission savings in the energy sector.

A report published by the Grantham Research Institute on Climate Change and the Environment, and ESRC Centre for Climate Change Economics and Policy at London School of Economics and Political Science concluded that there has been progress compared with hypothetical ‘business as usual’ global emissions pathways [49]. However a huge ‘gigatonne gap’ of 12 to 14 GtCO_2_e between the emissions pathway that would result from current ambitions and plans, including those goals outlined by the submitted Intended Nationally Determined Contributions (INDCs), and emission pathway that is consistent with a reasonable chance of achieving the planetary goal of staying below 2 °C temperature rise above pre-industrial levels [49, 50]. Increasing urban population, particularly in emerging economics and world’s most populous countries such as China and India, has created additional boost in annual global energy and infrastructure demand. In the contexts, energy substitution and material substitution effects associated with harvested wood products offer cleaner, safer and renewable energy source, and could be considered as an element of INDCs, and nationally appropriate mitigation actions.

## Methods

For our study we do not consider the problem of appropriate national accounting rules and reporting. Instead we use an emission balance approach, in which we extend the current scope of REDD+ and study by means of a scenario analysis (i) the reduction of forest C-stocks by logging residues, (ii) the transfer of carbon from the forest to the HWPs C-pool, and (iii) emission reductions by the production and use of timber as a replacement for energy-intensive materials and non-renewable energy sources. One solid cubic meter (m^3^) of wood is used as a standard unit for the analyses. As wood density is decisive for the further use of harvested timer, we included three different wood densities in the analyses, i.e. 500, 750 and 1000 kg/m^3^. These figures take into account the differences in wood densities found in tropical tree species and regions and reflect broad density classes. However, different forest with different species has a different capacity to store carbon and to increase carbon sequestration [51].

### Scenario analyses

Based on the assumptions presented above we developed three scenarios that quantify the carbon offsets by using wood for energy or as HWP. Emissions and mitigation potentials for both, the forest and timber sector are analyzed. Based on the standard unit of 1 m^3^ the substitution effects compared to natural gas, lignite, and residual fuel are presented for the three selected wood densities (i.e. 500, 750 and 1000 kg/m^3^). In addition to the extracted timber, the volume of logging residuals is considered as well. Logging residuals remain in the forest and result in emissions due to decay.

Values for logging and mills residues, emissions for energetic use, and displacement factor were taken from the literatures presented in the “Background” section of this paper.

#### Scenario 1. wood fuel

**Fig. 2:**
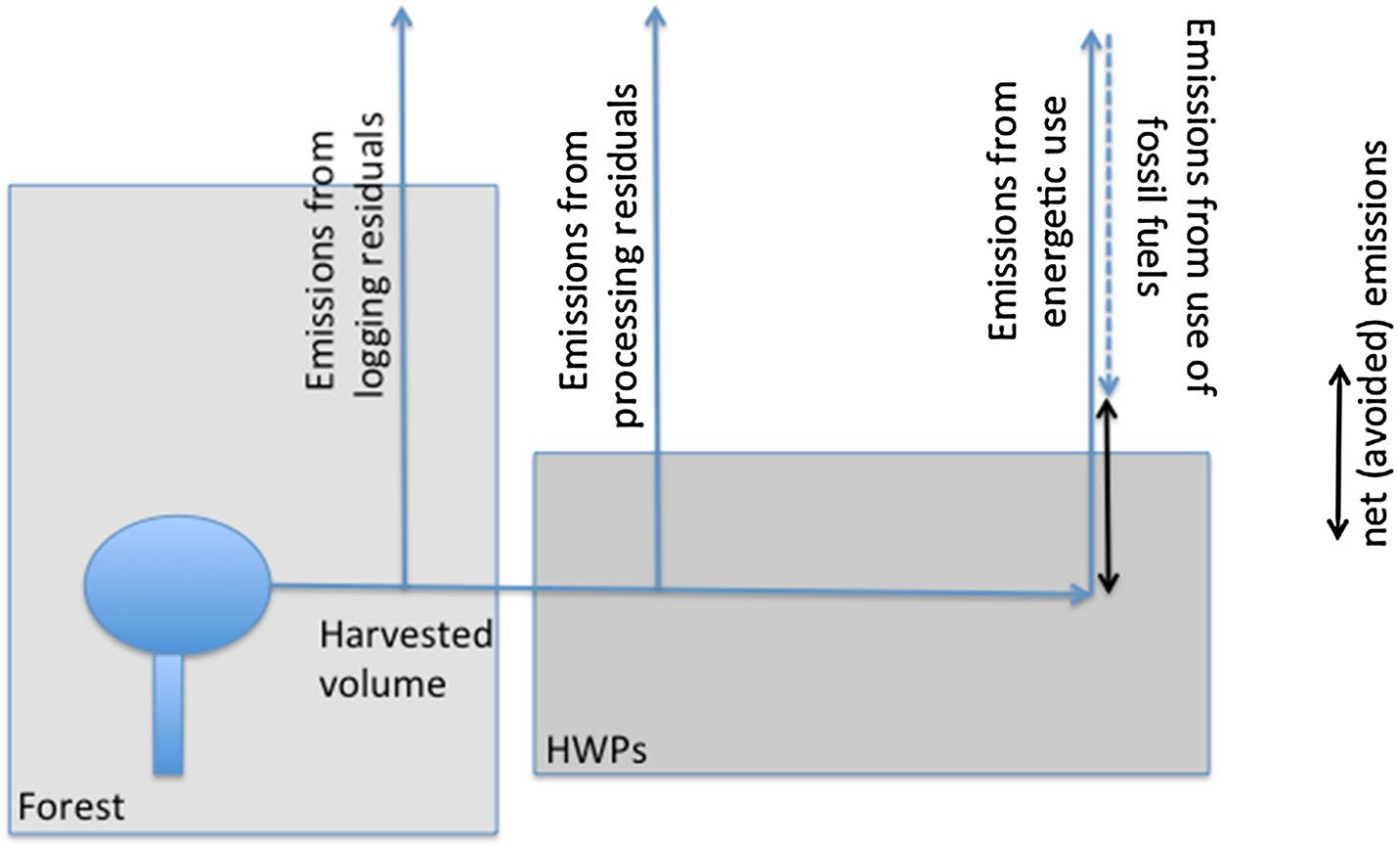
Scenario “woodfuel”

In scenario 1 the extracted timber is solely used for energy (Fig. 2). This represents a typical situation where harvesting of wood fuel leads to forest degradation. Wood fuels are still a major source of energy for people in Africa and Subtropical Asia, and wood fuel harvesting is the most important cause of forest degradation in African countries [52]. Wood fuel is typically showing smaller dimensions than logs for timber production and is associated with lower destruction by felling and skidding. Therefore we implement two sub-scenarios: in sub-scenario 1a, all biomass removed from the forest carbon stock is utilized for energy, and in sub-scenario 1b, logging losses are of the same amount as the extracted timber.

#### Scenario 2. harvested wood products

**Fig. 3:**
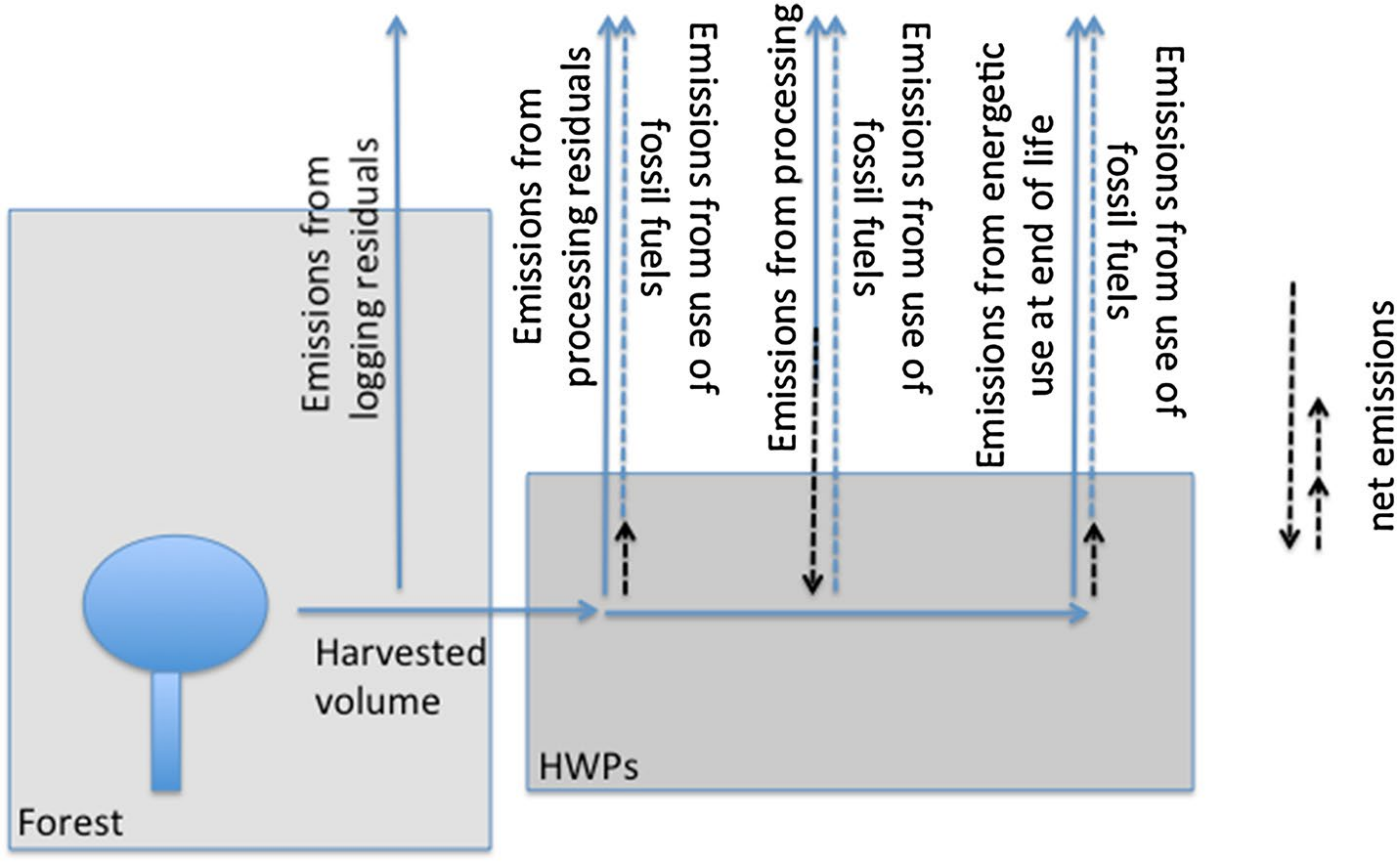
Scenario “HWP”

Scenario 2 focuses on the use of the extracted timber for construction timber as an example for HWPs (Fig. 3). Potential emission reductions are driven by the amount of logging and mill residuals, the displacement factor for HWPs, and the proportion of timber in HWPs that is used for energy at the end of the lifecycle of the HWP (Table 2). We implemented two conceptual structures of assumptions for the scenario analysis. A conservative approach is underlying sub-scenario 2a. This low efficiency scenario reflects a reserved attitude towards the potential emission reductions. Logging and mill residues are comparably large, no energetic use is assumed for mill residues and HWPs at the end of their lifecycle, and the displacement factor is low (Table 5). Sub-scenario 2b anticipates a more efficient use of timber. Reduced impact logging results in logging residues that are two times the amount of the extracted timber, mill residues amount to 40 % of the processed timber and are used for energy, 60 % of the timber in HWPs is used for energy at the end of the lifecycle, and the displacement factor is set to 2.1 (Table 6). We choose the two sub-scenarios to give insight in the range of emission reductions that is likely in the context of developing countries.

### Sensitivity analysis

Sensitivity analysis is an approach to assess the influence of the variance of input variables on the variance of the output variable [53]. The objective is to describe the influence of individual input variables on the resulting output. In a partial sensitivity analysis one input variable is selected and its values are changed while holding the values of the other input variables constant. The procedure is repeated for each input variable. We performed a partial sensitivity analysis using the input variables (1) logging residuals, (2) displacement factor, (3) energy from logging residuals, (4) energy from HWPs, and (5) type of fossil fuel for substitution minor and studied their effect on CO_2_ emissions. The ranges used for the individual input variables are given in Table 7.
